# A neuron-distributed DEK integrates ac4C modification and neuroinflammation in pathogenesis of Parkinson disease: evidence from Mendelian randomization, multi-omics and *in vitro* validation

**DOI:** 10.3389/fnins.2026.1950353

**Published:** 2026-09-01

**Authors:** Yuan Li

**Affiliations:** West China Hospital, West China School of Nursing, Sichuan University, Chengdu, Sichuan, China

**Keywords:** ac4C modification, DEK, machine learning, multi-omics, neuroinflammation, Parkinson’s disease

## Abstract

**Background:**

Epi-transcriptomic modifications, particularly N4-acetylcytidine (ac4C), and chronic neuroinflammation have emerged as pivotal players in the pathogenesis of Parkinson’s disease (PD). However, the specific molecular co-expression patterns linking ac4C RNA modification to neuronal inflammatory responses remains largely uncharted.

**Objective:**

This study aimed to decode the ac4C-neuroinflammation (AN)-associated molecular patterns in PD and to identify a neuron-specific central pathogenic and therapeutic factor.

**Methods:**

We integrated multi-omics analyses by using peripheral blood bulk transcriptomes (GSE18838, GSE49126, GSE22491, GSE6613, and GSE57475) and GWAS data from PD patients for identification of AN-related risk genes. Next, consensus clustering and 3 machine learning algorithms (LASSO, RF, and SVM-RFE) were applied for patient stratification, hub gene identification, and diagnostic modeling. Single-cell transcriptomic profiling of PD patients (GSE140231) was leveraged to map the cellular distribution and mechanistic roles of the hub gene within the substantia nigra (SN). An AI-driven active learning framework and the CTD database were utilized to screen therapeutic candidates targeting the hub gene, with binding affinities validated via molecular docking. *In vitro* experiments finally validated the expression of hub gene.

**Results:**

We pinpointed 7 AN-associated risk DEGs for PD patients, including HSP90AA1, DEK, LEF1, IRF2, BCL2L1, CFL1, and BCR, which effectively stratified PD patients into 2 distinct immune-molecular subgroups. DEK can be considered as neuron-distributed and up-regulated AN-associated central pathogenic factor for PD patients. The DrugReflector active learning framework identified BRD-K57589644 as a computationally prioritized compound warranting further investigation.

**Conclusion:**

This study establishes a novel AN-associated molecular patterns in PD, identifying DEK as a computationally identified neuron-specific factor associated with ac4C modification and neuroinflammatory cascades for PD patients.

## 1 Introduction

Parkinson’s disease (PD) represents a progressive neurodegenerative condition characterized neuropathologically by a pronounced depletion of dopaminergic neurons within the substantia nigra (SN), a feature that affects an estimated 1% of individuals aged 60 years and older globally (Hayes, 2019). The pathophysiology of PD extends not only includes classic motor symptoms, such as bradykinesia, rigidity, and tremor, but also encompass systemic dysfunctions, including a sustained, low-grade chronic neuroinflammation (Elsworth, 1996). Chronic inflammation was activated by microglia and infiltrating immune cells, is a hallmark of PD pathology and is intimately tied to the degeneration of dopaminergic neurons (Sveinbjornsdottir, 2016).

Beyond the well-established landscape of DNA methylation and histone acetylation, the field of RNA epi-transcriptomics has emerged as a critical frontier in gene regulation (Yang et al., 2023). Among the myriads of RNA modifications, N4-acetylcytidine (ac4C) acetylation has recently garnered considerable attention for its capacity to govern both the stability of mRNA transcripts and the fidelity of translational elongation (Wang H. et al., 2025). Unlike the extensively studied N6-methyladenosine (m6A) modification, ac4C is primarily catalyzed by the N-acetyltransferase 10 (NAT10) complex, which functions in concert with co-activators such as CBP and p300, epigenetic writers also known for their roles in histone acetylation (Liu H. et al., 2024). This enzymatic machinery not only introduces ac4C marks onto coding and non-coding RNAs but also orchestrates the RNA metabolic reprogramming essential for cellular stress responses and functional adaptation (Wang Q. et al., 2025). In the central nervous system, ac4C modification has been implicated in neural development, synaptic plasticity, and the regulation of intrinsic neuronal survival pathways (Xu et al., 2025). Emerging evidence further suggests that NAT10-mediated ac4C deposition can directly influence the expression of pro-inflammatory cytokines and chemokines by modulating the stability of their corresponding transcripts (Zhang et al., 2023). However, while ac4C dysregulation is increasingly linked to neurodegenerative conditions such as Alzheimer’s disease, its precise functional contribution to the glial-neuronal inflammatory crosstalk within the substantia nigra (SN) of Parkinson’s disease (PD) remains largely uncharted.

To address this knowledge gap, the present investigation deployed an integrative end-to-end analytical pipeline, combining causal inference via Mendelian randomization (MR), machine learning, single-cell mapping, and *in vitro* functional validation to systematically define the ac4C and neuroinflammation (AN)-associated molecular patterns and its central neuronal driver in PD.

## 2 Materials and methods

### 2.1 Bulk data source and pre-processing

Four peripheral blood bulk transcriptomic datasets of PD patients (GSE18838, GSE49126, GSE22491, and GSE6613) were obtained from the GEO database via GEOquery package in R (Wang S. et al., 2025). GSE18838 (detected on GPL5172) included 11 healthy controls and 19 PD samples (Wei et al., 2025). GSE49126 (detected on GPL4133) included 20 controls and 30 PD samples (Xing et al., 2022). GSE22491 (detected on GPL6480) included 8 controls and 10 PD samples (Liu T. et al., 2024). GSE6613 (detected on GPL6480) included 25 controls and 47 PD samples (Yuan et al., 2020). GSE57475 (GPL6947) contained 93 PD samples and 49 healthy controls (Zhou et al., 2018). All datasets were normalized using the limma R package (Ritchie et al., 2015). The ac4C modification-related and neuroinflammation-related gene lists were curated from the GeneCards database with a relevance threshold > 1 (Zhou et al., 2026). These 2 sets were intersected to generate the AN-associated gene list.

### 2.2 Differentially expressed analysis

Differential expression analysis (DEGs) was performed on the GSE18838 dataset using the limma R package (Ritchie et al., 2015). Significant DEGs were defined based on thresholds of logFC greater than 1 and *p* value less than 0.05 (Ritchie et al., 2015). Visualization was conducted via ggplot2 and ComplexHeatmap packages in R (Gustavsson et al., 2022; Gu, 2022). Subsequently, the DEGs were intersected with the AN-associated gene list to identify AN-related DEGs (Supplementary Table S4). Functional enrichment (KEGG and GO analysis) was performed via clusterProfiler package in R using MSigDB KEGG and GO gene sets (Yu et al., 2012).

### 2.3 MR analysis

Causal relationships between AN-associated DEGs and PD were assessed using MR (Liu et al., 2025). GWAS summary statistics (ieu-b-7: 33,674 PD cases, 449,056 controls, and 17,891,936 SNPs) were retrieved from the IEU Open GWAS database (Liu et al., 2025). The AN-associated DEGs were designated as exposure instrumental variables (Liu et al., 2025). After harmonizing exposure and outcome alleles using the TwoSampleMR R package, reverse causality and pleiotropy were evaluated using the MR-Egger intercept and heterogeneity tests (Wang E. et al., 2025). The specific MR method applied was the inverse variance weighted (IVW) method using random-effects models (Wang E. et al., 2025). To assess the robustness of the causal estimates, we performed multiple sensitivity analyses, including MR-Egger regression to test for directional pleiotropy, weighted median estimator to provide consistent causal estimates even when up to 50% of the instruments are invalid and leave-one-out sensitivity analyses to evaluate whether any single SNP drove the causal association (Wang E. et al., 2025). For each of the 7 AN-associated risk genes, we reported the number of SNPs used as instrumental variables (range from 3 to 12 SNPs per gene), the proportion of variance explained (R^2^), and the F-statistic for each instrument (Wang E. et al., 2025). Heterogeneity was assessed using Cochran’s Q statistic, and the MR-Egger intercept was used to test for horizontal pleiotropy (*p* > 0.05 for all genes) (Wang E. et al., 2025). F SNPs with a *p*-value less than 0.05 in the IV method were considered statistically significant risk factors (Wang E. et al., 2025).

### 2.4 Machine learning for diagnostic model construction

In GSE49126, 3 complementary machine learning algorithms, including LASSO logistic regression (generated by glmnet package in R), Support Vector Machine-Recursive Feature Elimination (generated by e1071 package in R), and Random Forest (generated by randomforest package in R), were applied to the GSE49126 training dataset based on the AN-associated risk DEGs (Xu et al., 2023). The genes consistently selected by all 3 algorithms were defined as the ultimate hub gene (Xu et al., 2023). Nomograms were constructed via the rms package in R. Diagnostic performance (ROC and PR curves) were examined in GSE49126, GSE22419 and GSE6613 by using the pROC package in R (Liu et al., 2023). Diagnostic accuracy(nomogram and calibration analysis) was examined by using rms package in R (Liu et al., 2023). Immune infiltration correlations with the hub gene were assessed using the CIBERSORT algorithm in R (Wang et al., 2022). Single-gene GSEA analysis was conducted by clusterProfiler package using MSigDB hallmark gene sets (Ma, 2026). Hub gene chromatin localization was quantified by circlize package in R (Chen et al., 2026).

### 2.5 Molecular subtyping analysis

Deployment of the ConsensusClusterPlus R package on the 93 PD patient samples from GSE57475 was executed to perform consensus clustering in R (Zhang and Song, 2026). Determination of the optimal cluster count was guided by inspection of the CDF curves (Zhang and Song, 2026). Subsequently, assessment of immune compositional differences and pathway enrichment discrepancies between the identified subgroups was undertaken via the CIBERSORT algorithm and GSEA (using clusterProfiler with reference to MSigDB KEGG and GO signatures), respectively (Zhang and Song, 2026). Additionally, the relative transcript levels of the AN-associated risk DEGs were examined to compare their expression profiles across the defined molecular subgroups (Zhang and Song, 2026).

### 2.6 Single-cell transcriptomic analysis

Procurement of the GSE140231 scRNA-seq dataset (derived from the SN of 7 PD patients) was achieved via the GEO repository, with subsequent analytical processing carried out through the Seurat R package (Chen et al., 2026). Stringent quality control criteria were enforced, excluding cells based on total gene counts, RNA counts, or a mitochondrial gene ratio exceeding 15% (Chen et al., 2026). Non-linear dimensionality reduction was accomplished via UMAP and t-SNE projections (Chen et al., 2026). Cell type identities were assigned using the scMayoMap R package in R (Chen et al., 2026). Interrogation of intercellular communication networks and metabolic pathway landscapes was undertaken via the CellTalker and scMetabolism packages in R, respectively (Chen et al., 2026). Reconstruction of neuronal pseudotime trajectories, alongside the spatiotemporal expression patterns of DEK, was performed via the Monocle2 algorithm in R (Chen et al., 2026). Following the spatial mapping of the AN-associated hub gene via the ggplot2 package, the scTenifoldKnk algorithm was deployed to computationally simulate gene depletion specifically within the targeted cellular population, and the downstream functional repercussions of this perturbation were subsequently evaluated through KEGG and GO enrichment analyses, carried out via the clusterProfiler package in R utilizing gene sets obtained from the MSigDB database (Chen et al., 2026). Additionally, the interactome of the dysregulated molecular entities resulting from the *in silico* knockout was interrogated through the GENEMANIA platform (Chen et al., 2026).

### 2.7 Therapeutic agent estimation and molecular docking

Utilization of the DrugReflector active learning framework was undertaken to interrogate the cMAP database, leveraging the GSE49126-derived gene signature for the identification of compounds with PD reversal potential (Chen et al., 2026). The algorithm computed a reversal probability based on the negative cosine similarity between the disease signature and the drug perturbation profiles (Chen et al., 2026). To prioritize high-confidence candidates, a screening threshold of reversal probability >0.10 was applied, and compounds were subsequently ranked by their probability scores (Chen et al., 2026). The compound exhibiting the highest reversal probability was selected as the top candidate (Chen et al., 2026). In addition, the CTD repository was queried to search for natural products targeting hub gene (Zeng et al., 2026). The search strategy utilized the target gene symbol DEK and filtered chemical-gene interactions based on the following criteria, such as a curated interaction score threshold >5, and at least 2 independent scientific references supporting the chemical-gene association in the CTD literature (Zeng et al., 2026). Quercetin and Catechin were identified as the top-ranked natural compounds meeting these criteria and were thus selected for subsequent molecular docking simulations (Zeng et al., 2026). Molecular docking simulations were performed using AutoDock Vina (Yang and Hu, 2026). The DEK target structure (PDB ID: 8KD1) was retrieved from the PDB, while ligand structures were sourced from PubChem (Quercetin CID: 5280343; Catechin CID: 9064) (Yang and Hu, 2026). Protein and ligand pre-processing, including water removal, polar hydrogen addition, and Gasteiger charge assignment, was conducted via AutoDock Tools (Yang and Hu, 2026). The docking grid box was defined as 20 Å × 20 Å × 20 Å with a 0.375 Å spacing, centered at the geometric centroid of the known active pocket (Yang and Hu, 2026). An exhaustiveness of 8 was applied, with 10 conformations generated per ligand (Yang and Hu, 2026). The binding pose with the lowest Vina score (kcal/mol) was selected, and intermolecular interactions (hydrogen bonds and hydrophobic contacts) were visualized using PyMOL software (Yang and Hu, 2026).

### 2.8 *In vitro* assay validation

To establish an *in vitro* PD model, SH-SY5Y cells procured from SUNCELL company (China) were maintained in DMEM supplemented with 10% FBS at 37 °C under 5% CO₂, and subsequently subjected to 200 μM MPP^+^ (Sigma-Aldrich, #D048) for 24 h (Ma, 2026). Total RNA was isolated via TRIzol reagent (TaKaRa) and subsequently converted into cDNA using HiScript II Q RT SuperMix. Quantitative real-time PCR was conducted on a StepOnePlus platform with SYBR Green MasterMix, utilizing β-actin as the internal reference. The specific oligonucleotide primers applied in the amplification reactions are itemized below:

DEK:

F:5′- AACTGCTTTACAACAGGCCAG-3′.

R:5′- ATGGTTTGCCAGAAGGCTTTG-3′.

β-actin:

F:5′- CATGTACGTTGCTATCCAGGC-3′.

R: 5’-CTCCTTAATGTCACGCACGAT-3’.

### 2.9 Statistical analysis

All statistical computations were carried out using R (version 4.2.2) and GraphPad Prism (version 9.0). Normality of continuous variables was assessed using the Shapiro–Wilk test; normally distributed data were compared using Student’s t-test, while non-normally distributed data were analyzed using the Wilcoxon rank-sum test. For multiple comparisons, one-way ANOVA with Tukey’s post-hoc test was applied. Spearman’s correlation coefficient was utilized to evaluate associations between variables. For enrichment analyses (GO, KEGG, and GSEA), the Benjamini-Hochberg method was applied to control the FDR, with FDR < 0.05 considered significant. The sample sizes for the *in vitro* experiments (n = 3 independent biological replicates per group) were determined based on previous studies using the SH-SY5Y MPP^+^ model, and a post-hoc power analysis indicated that the study had 80% power to detect a 2-fold difference in gene expression at α = 0.05. A two-tailed *p*-value of less than 0.05 was considered to indicate statistical significance.

## 3 Results

### 3.1 Identification of AN-associated DEGs for PD patients

**Figure 1:**
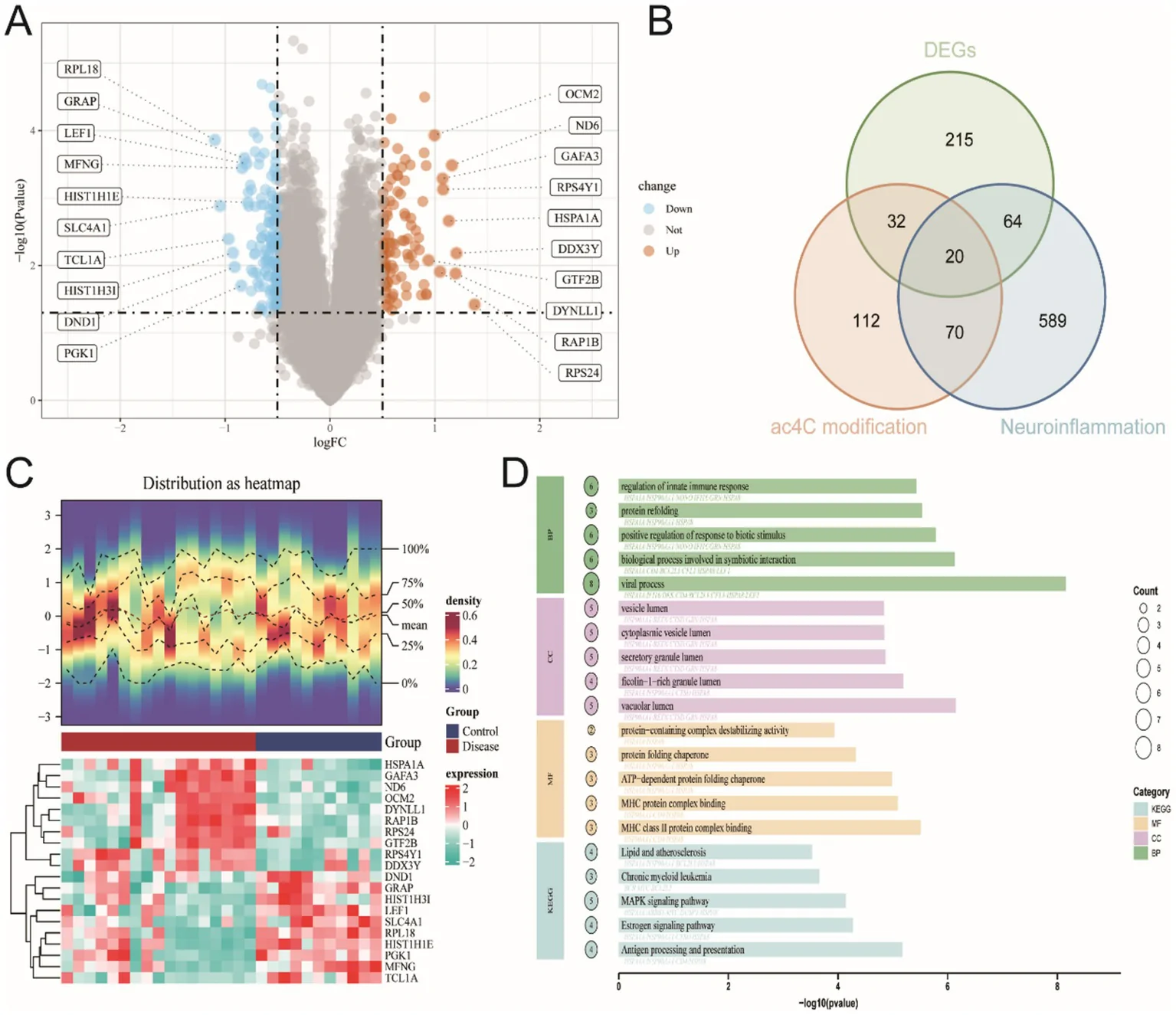
Delineation of AN-associated DEGs in PD. **(A)** Differential expression between PD patients and healthy controls from the GSE18838 cohort was visualized via a volcano plot. **(B)** The overlap between the complete DEG repertoire and the AN-associated gene lists was depicted by a Venn diagram. **(C)** Hierarchical clustering of the 20 identified AN-associated DEGs revealed distinct expression patterns across the samples. **(D)** GO and KEGG functional enrichment profiles of the 20 AN-associated DEGs.

Interrogation of the GSE18838 dataset via the Limma pipeline delineated 101 upregulated and 135 downregulated DEGs in PD relative to controls. Subsequent intersection of these transcripts with the AN-related gene list yielded 20 core AN-associated DEGs (Figures 1A–C). Functional enrichment profiling via KEGG and GO analyses subsequently revealed that these 20 genes were predominantly enriched in neuroinflammatory cascades and epigenetic regulatory pathways (Figure 1D).

### 3.2 AN-associated risk DEGs identification for PD patients

**Figure 2:**
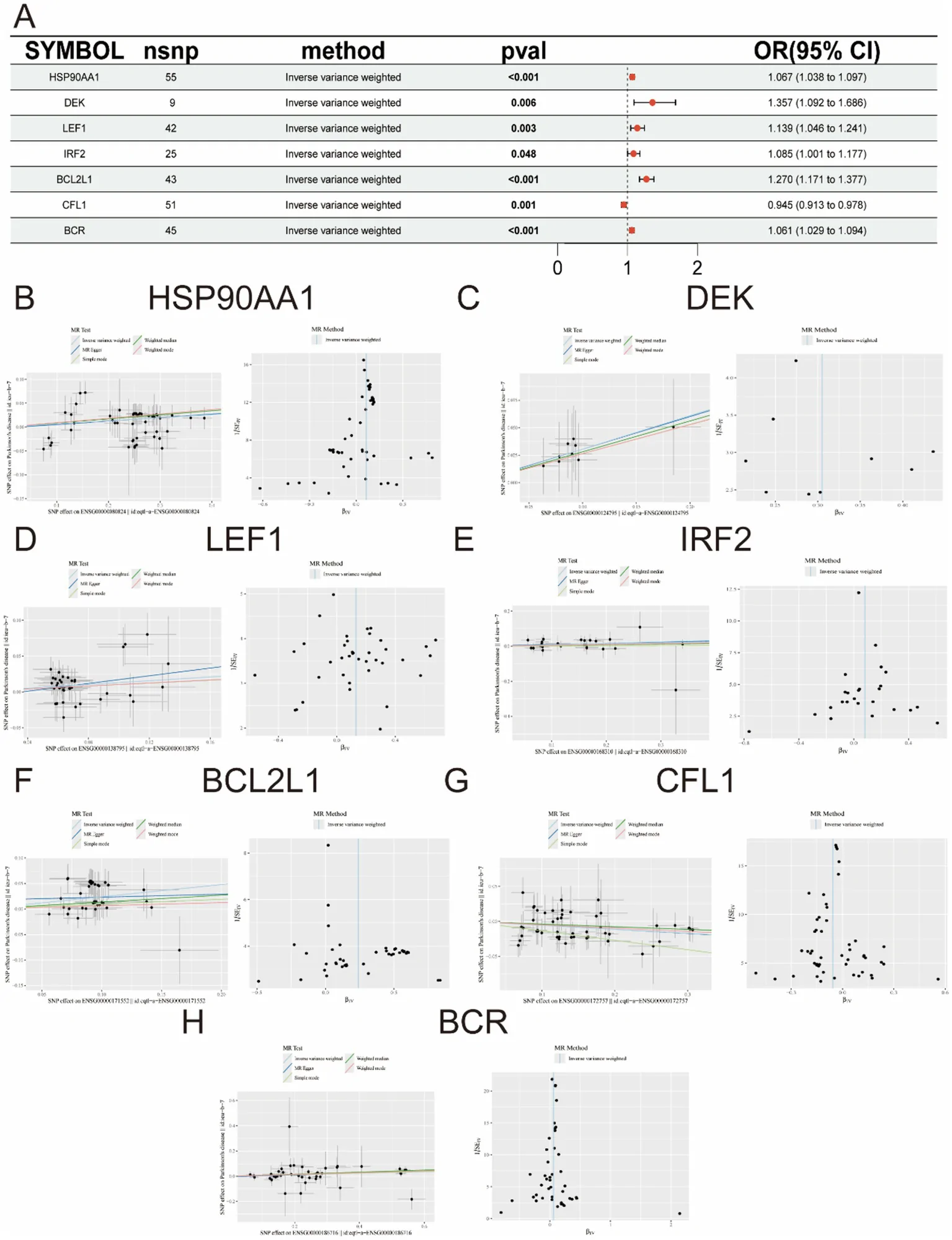
MR pinpoints AN-associated risk DEGs in PD. **(A)** Forest plots and scatter plots displaying the causal associations between the 7 AN-associated risk DEGs and PD. **(B-H)** Scatter plots of the 7 AN-associated risk DEGs by MR analysis.

To filter out spurious associations and infer causality, the 20 AN-associated DEGs were exposed to MR analysis using the PD GWAS data. Following rigorous heterogeneity, pleiotropy, and Steiger directional tests, 7 AN-associated risk DEGs, including HSP90AA1, DEK, LEF1, IRF2, BCL2L1, CFL1, and BCR, were identified as having significant causal associations with PD pathogenesis (Figures 2A–H, Supplementary Figures S1A–G, and Supplementary Tables S1–S3).

### 3.3 AN-associated molecular subgroup identification for PD patients

**Figure 3:**
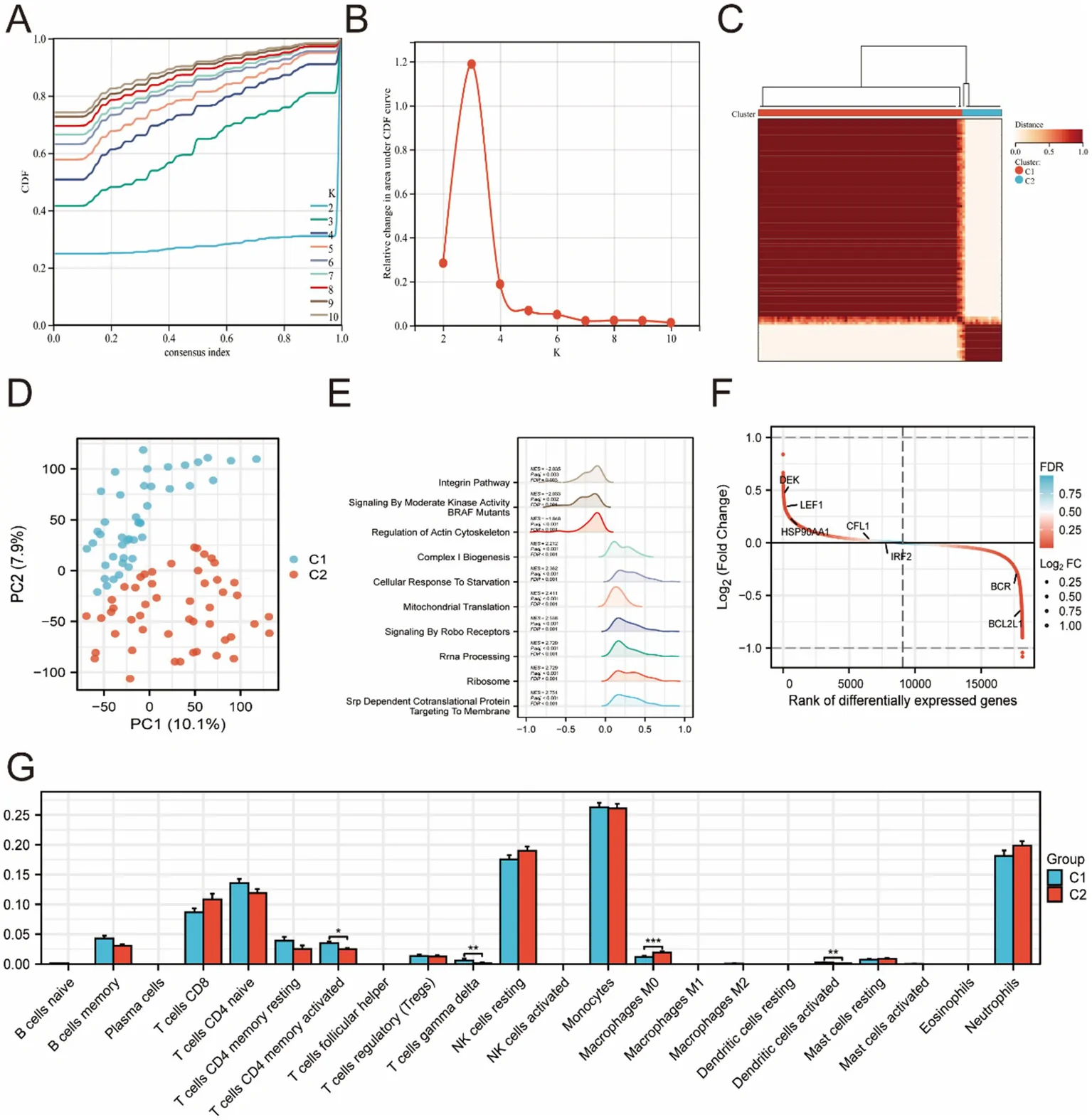
AN-associated molecular subgroups in PD. **(A–C)** Consensus clustering matrix heatmaps and CDF curves guiding the optimal stratification of 93 PD patients into 2 molecular subgroups (C1 and C2) based on the 7 AN-associated risk genes. **(D)** PCA plots confirming the spatial separation of C1 and C2 subgroups. **(E)** GSEA enrichment plots revealing distinct pathway activation patterns in C1 and C2. **(F)** AN-associated DEGs expression between C1 and C2 group. **(G)** CIBERSORT analysis demonstrating significant differences in immune cell infiltration.

Based on the 7 risk DEGs, consensus clustering was applied to the 93 PD samples in GSE57475. This analysis robustly divided the cohort into two distinct molecular subgroups, designated C1 and C2 (Figures 3A–D). Immune landscape analysis revealed profound heterogeneity between these groups; notably, the C2 subgroup exhibited significantly higher infiltration of monocytes and regulatory T cells (Figures 3E–G). This stratification suggests that AN-related signatures could serve as a foundation for personalized precision medicine in PD.

### 3.4 AN-associated hub gene identification for PD patients

**Figure 4:**
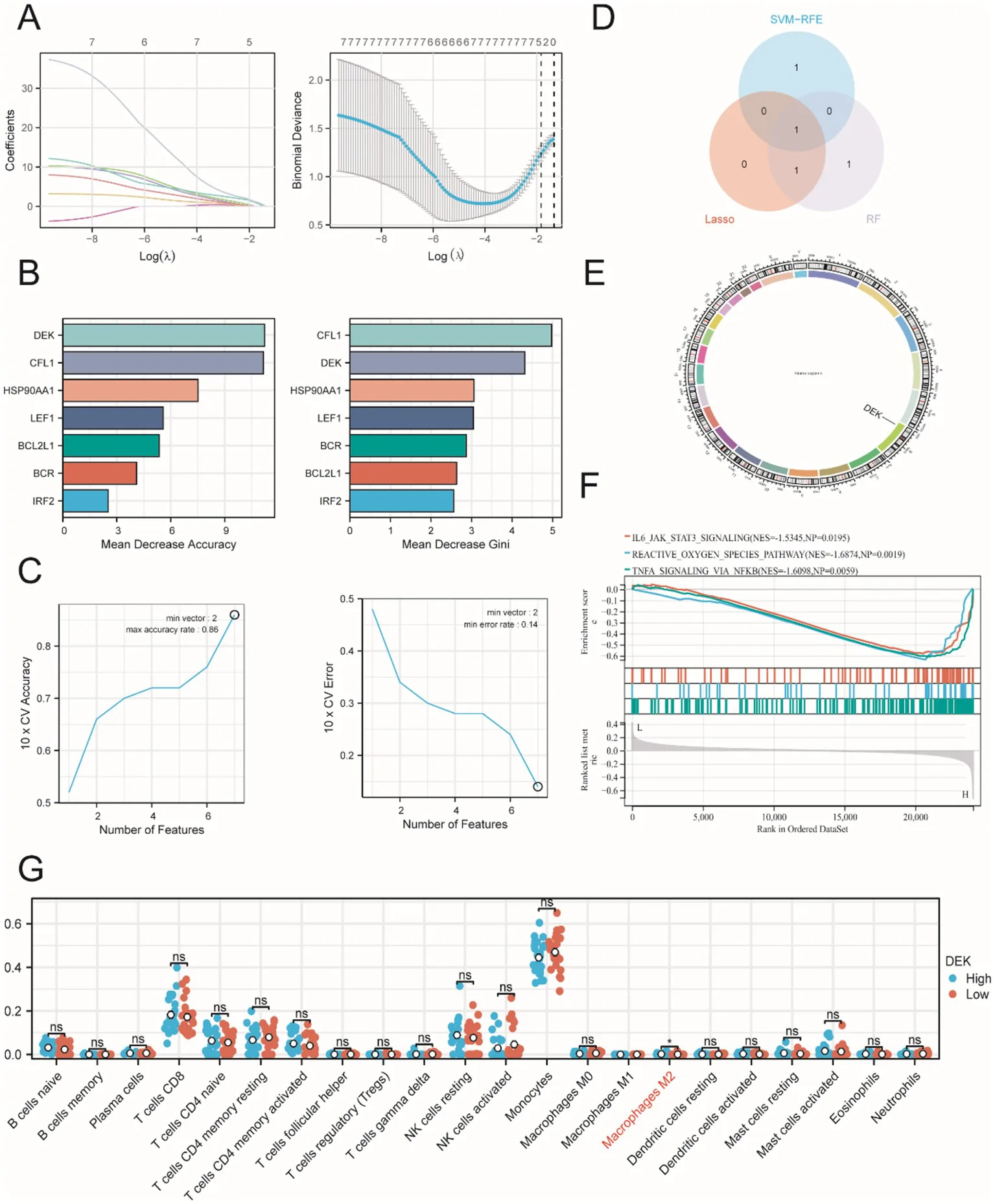
Identification of DEK as the AN-associated hub gene in PD via machine learning in PD. **(A)** Coefficient profiles from LASSO logistic regression, optimized through 10-fold cross-validation. **(B)** Variable importance ranking generated by RF based on the Mean Decrease Gini and Accuracy metrics. **(C)** Accuracy and error rate trajectories across feature subsets during SVM-RFE. **(D)** Venn diagram depicting the intersection of the three algorithms, converging uniquely on DEK. **(E)** Circos plot illustrating the chromosomal positioning of DEK. **(F)** Single-gene GSEA enrichment profile demonstrating the engagement of DEK in T-cell receptor signaling. **(G)** CIBERSORT-based correlation evaluation between DEK expression and immune infiltration in the PD cohort.

Deployment of three complementary machine learning algorithms, including LASSO, RF, and SVM-RFE was undertaken on the GSE49126 training cohort to narrow down the central driver from the 7 identified risk genes (Figures 4A–C). Cross-validation across all three methods yielded a consensus outcome that converged upon a single transcript, identified as DEK (Figures 4D,E). Subsequent functional profiling via single-gene GSEA and immune deconvolution via CIBERSORT revealed that DEK was intimately involved in the orchestration of inflammatory cascades throughout PD progression (Figures 4F,G).

### 3.5 AN-associated diagnostic model construction for PD patients

**Figure 5:**
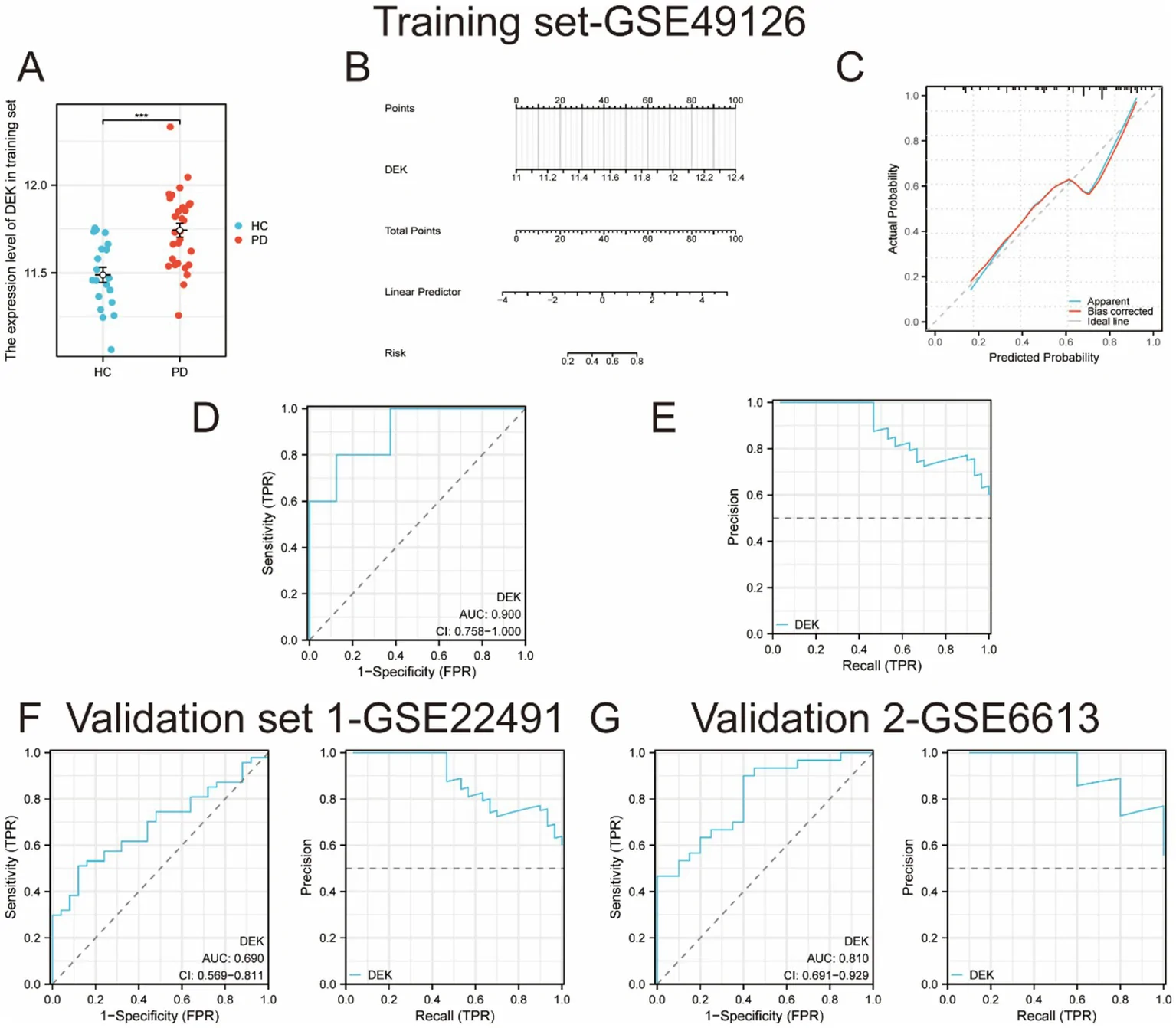
Diagnostic performance of DEK in PD. **(A)** Box plot showing the significantly upregulated expression of DEK in PD patients compared to controls in the GSE49126 training set. **(B,C)** Nomogram and calibration curve constructed based on DEK expression, demonstrating favorable predictive accuracy. **(D–G)** ROC and PR curves evaluating DEK diagnostic performance in the training and 2 independent validation cohorts.

In the GSE49126 training set, DEK was significantly upregulated in PD patients compared to controls (Figure 5A). To verify its translational potential, ROC and PR curves were constructed in training set and 2 independent validation cohorts (GSE22491 and GSE6613), both revealing robust diagnostic accuracy and efficacy, suggesting DEK as a highly promising liquid biopsy biomarker for PD (Figures 5B–G).

### 3.6 Cell chat and metabolic rewriting in SN microenvironment for PD patients

**Figure 6:**
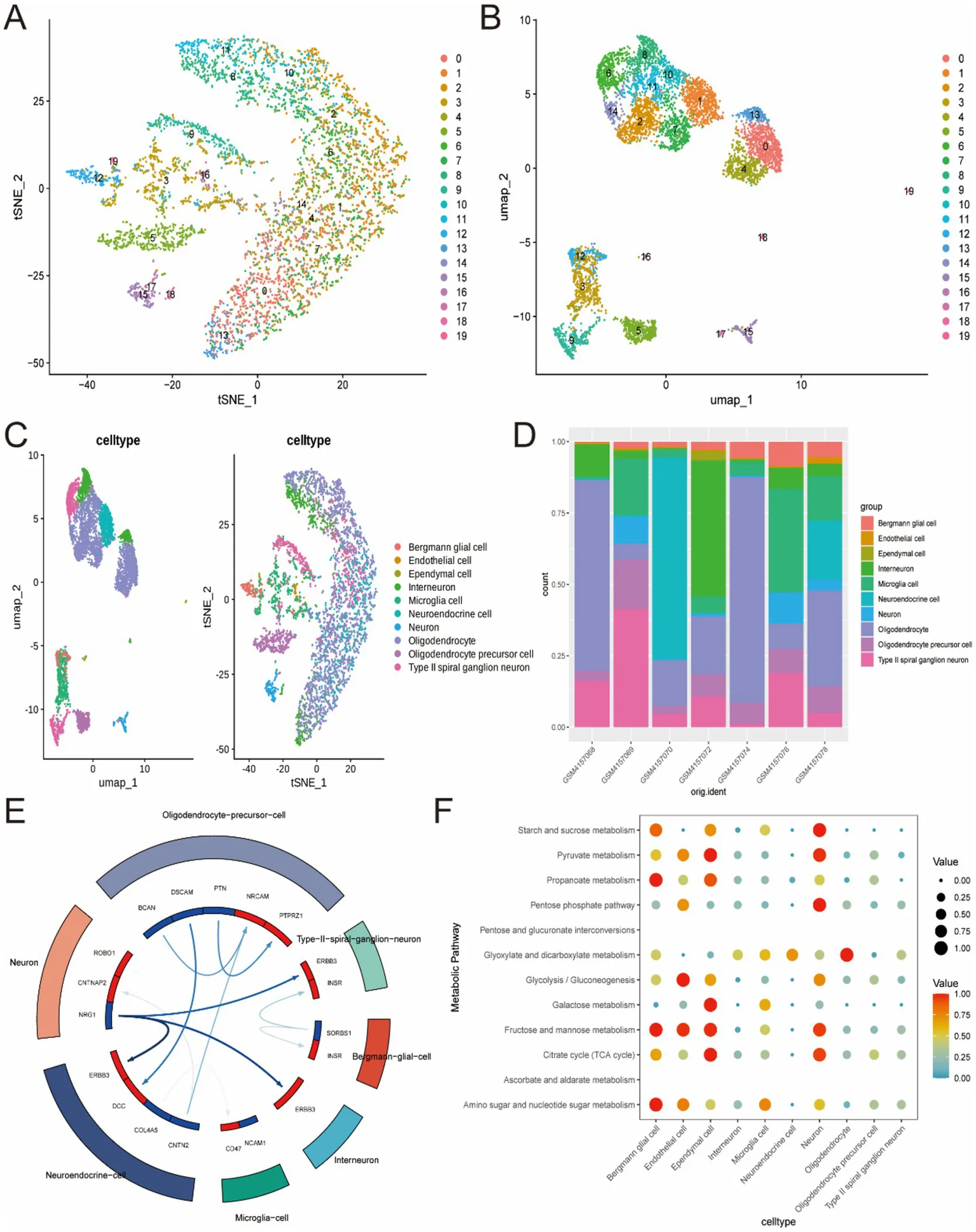
Single-cell landscape of DEK in the PD SN. **(A,B)** UMAP and t-SNE visualization of the 19 cell clusters. **(C,D)** UMAP and t-SNE plots annotating the 10 major cell types within the PD SN microenvironment and corresponding cell proportion analysis. **(E)** CellTalker network analysis revealing complex intercellular communication patterns among the largest cell populations. **(F)** Dot plot displaying metabolic pathway heterogeneity across the 10 cell types.

Single-cell analysis of the GSE140231 dataset identified 19 cell clusters and 10 distinct cell types within the PD SN microenvironment (Figures 6A–C, Supplementary Figures S2A–F). Oligodendrocytes, interneurons, and type II spiral ganglion neurons constituted the largest proportions (Figure 6D). CellTalker analysis revealed intricate intercellular communication patterns among these populations, while metabolic profiling indicated significant heterogeneity in energy metabolism (Figures 6E,F).

### 3.7 AN-associated hub gene molecular mechanisms in neuron of SN for PD patients

**Figure 7:**
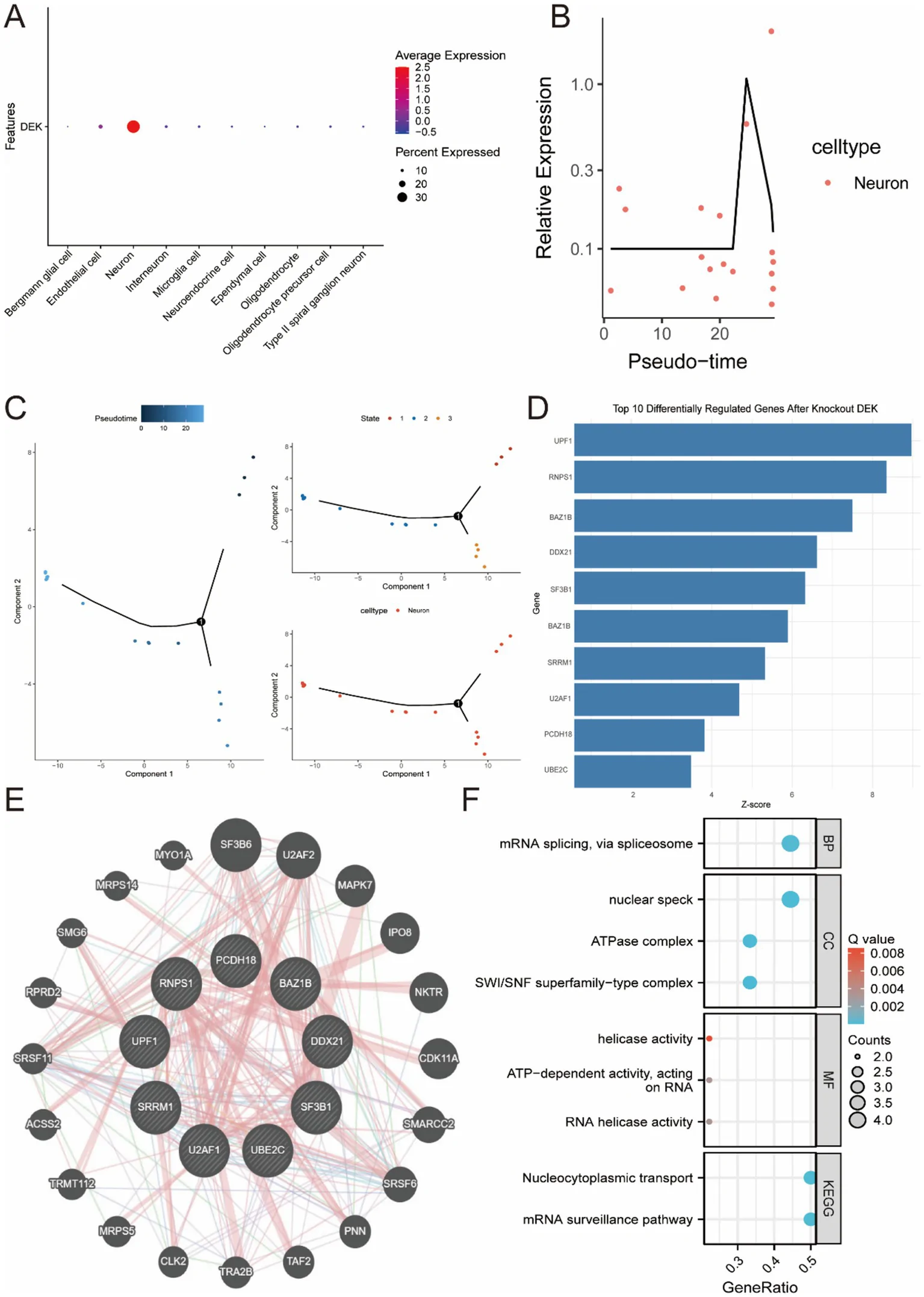
AN-associated molecular signature in neuron of SN for PD patients. **(A)** Feature plot confirming the predominant localization of DEK within the neuronal cluster. **(B,C)** Monocle2 pseudotime trajectory of neuronal differentiation and the dynamic expression of DEK along the neuronal pseudotime trajectory. **(D–F)** Computational KO of DEK in neuron and corresponding functional enrichment analysis.

Crucially, feature plots confirmed that DEK was predominantly localized to neurons, and Monocle2 pseudotime analysis revealed that DEK expression was closely associated with the later differentiation stages of neuronal development (Figures 7A–F). Besides, computational KO of DEK in neurons computationally predicted involvement in the regulation of functions related to neuroinflammation and mRNA epigenetic modification.

### 3.8 Therapeutic enrichment targeting AN-associated hub gene for PD patients

**Figure 8:**
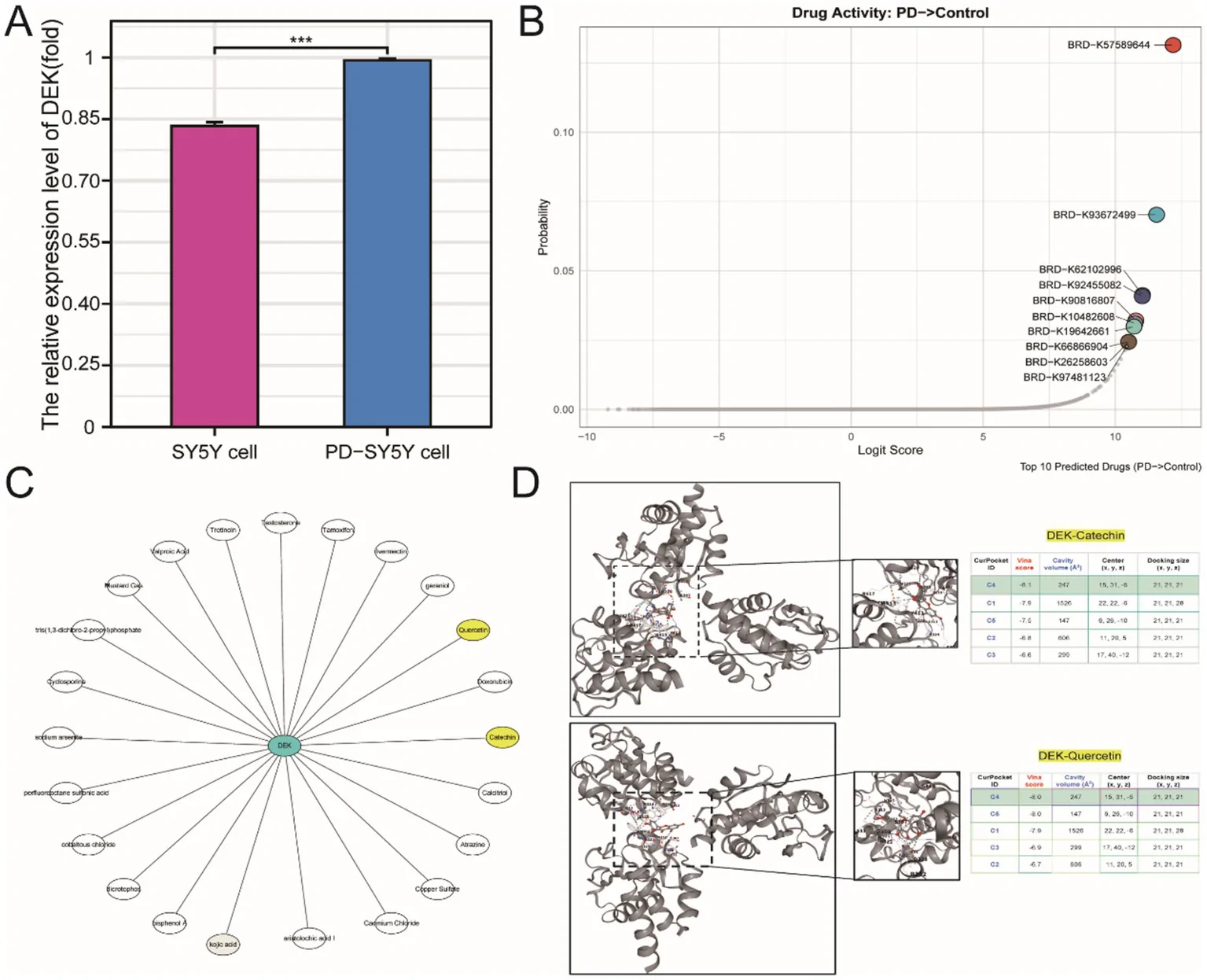
*In vitro* validation of DEK upregulation and therapeutic screening for PD. **(A)** Q-RT-PCR analysis confirming significantly elevated DEK protein levels in MPP + -treated SH-SY5Y cells compared to normal controls. **(B)** DrugReflector active learning screening identifying BRD-K57589644 as the optimal agent for reversing the PD transcriptomic signature. **(C)** CTD database enrichment identifying the natural compounds Quercetin and Catechin as potential DEK-targeting agents, along with their chemical structures. **(D)** 3D molecular docking models illustrating the favorable binding conformations of Quercetin and Catechin with the DEK protein.

In the MPP + -treated SH-SY5Y cell model, both q-RT-PCR analyses confirmed a significant upregulation of DEK mRNA and protein levels compared to the normal control cells (Figure 8A). The DrugReflector active learning framework identified BRD-K57589644 as the optimal agent for reversing the PD status (Figure 8B). Simultaneously, CTD database screening pinpointed Quercetin and Catechin as potential DEK-targeted natural compounds (Figure 8C). Molecular docking predicted stable and favorable binding conformations for both natural compounds with the DEK protein, supporting their potential therapeutic role (Figure 8D).

## 4 Discussion and conclusion

The present investigation establishes a previously unrecognized mechanistic link between epi-transcriptomic ac4C modifications and neuroinflammatory cascades in the pathogenesis of PD. Through a rigorous multi-omics pipeline anchored by MR, single-cell resolution, machine learning, and therapeutic screening, we identified DEK emerges as a central, neuron-specific regulatory central pathogenic and therapeutic target that integrates the AN-associated molecular patterns. While DEK has been historically recognized for its roles in DNA architecture, chromatin remodeling, and RNA splicing, its involvement in neurodegenerative contexts, particularly in crosstalk between RNA modifications and inflammatory responses, has remained largely uncharted (Bai et al., 2023; Shen et al., 2025).

We identified the upregulation of DEK identified in peripheral blood and SN neurons aligns closely with the concept of systemic immune activation in PD. Mechanistically, the CBP and p300 complex, the primary epigenetic writers for ac4C modifications, interacts closely with DEK to regulate chromatin accessibility and the transcription of inflammatory mediators (Zhang et al., 2024). The increased expression of DEK in response to ac4C dysregulation may potentiate the NF-κB signaling pathway, thereby exacerbating the local neuroinflammatory milieu and contributing to the progressive loss of dopaminergic neurons (Schiffers and Oberdoerffer, 2024). This study provides the first evidence that DEK may act as a phenotypic switch between epigenetic RNA modifications and neuronal vulnerability in PD (Jiao et al., 2025). Clinically, in our study, the robust diagnostic capacity of DEK across multiple independent cohorts, coupled with its distinct expression in neuronal subtypes, positions it as a compelling non-invasive liquid biopsy biomarker. Furthermore, the identification of BRD-K57589644 through an AI-driven active learning framework, alongside the natural compounds Quercetin and Catechin, offers a multi-layered therapeutic strategy to disrupt the AN-related molecular signature. Quercetin and Catechin, well-known flavonoids with established anti-inflammatory and neuroprotective properties, now possess a computational rationale for targeting DEK specifically (Deepika and Maurya, 2022; Musial et al., 2020).

In conclusion, this study provides an integrative, mechanistic framework linking ac4C epigenetic modification with neuroinflammation in PD, elevating DEK to a central neuron-specific target with dual diagnostic and therapeutic potential, thereby paving the way for precision medicine interventions for PD patients. Several limitations should be noted. First, it must be explicitly acknowledged that the diagnostic model and molecular subgroup identification presented in this study were entirely derived from public transcriptomic cohorts. While these datasets provide valuable insights into the molecular landscape of PD, their retrospective and cross-sectional nature introduces inherent limitations. In future studies, prospective, multi-center cohort studies with standardized clinical data collection are urgently required to validate the diagnostic performance of the DEK-based nomogram and to confirm the clinical utility of the identified molecular subgroups. Second, integrating our transcriptomic findings with readily accessible clinical parameters to construct a comprehensive, multi-modal predictive framework would significantly enhance its practical applicability in real-world clinical settings. Third, applying advanced batch-effect correction algorithms or harmonization techniques across multi-platform datasets will be essential to further improve the robustness and reproducibility of the DEK-based stratification before any clinical translation can be considered. Finally, longitudinal follow-up studies are needed to determine whether the C1 and C2 subgroups correlate with distinct trajectories of disease progression or differing responses to current antiparkinsonian therapies. Besides, it is important to critically acknowledge that, despite the robust computational and genetic associations established in this study, direct prior evidence supporting a specific mechanistic co-expression pattern linking ac4C modification, DEK function, and neuroinflammatory cascades in PD remains entirely lacking. Our findings, primarily derived from MR, transcriptomic profiling, and single-cell spatial mapping, provide strong correlational evidence. However, they do not definitively demonstrate that DEK protein is directly modified by ac4C or that its subsequent regulatory effects on neuroinflammation are explicitly ac4C-dependent. Future studies employing acRIP-seq for identification of ac4C sites on DEK mRNA or protein and CBP and p300-specific inhibitors or knockdown experiments in neuronal models are critically required to dissect the precise molecular architecture of this proposed axis. It should be noted that the single-cell dataset GSE140231 contains only 7 PD patient samples without healthy controls. Therefore, the observed cellular distribution and pseudotime trajectories of DEK reflect patterns within the PD context and cannot be attributed specifically to disease-associated changes. The findings from this dataset should be interpreted as descriptive of the PD neuronal landscape rather than comparative. Future studies should combined advanced AI technologies in higher resolution single-cell and spatial data of PD patients, coupled with pre-clinical studies, dynamically tracing AN-associated molecular pattern in neuron for the pathogenesis of AD patients. Similarly, the virtual knockout results are computational predictions based on gene regulatory network inference and should not be interpreted as functional evidence. Direct experimental validation through gene knockdown or knockout in neuronal models is required to confirm these predictions. Finally, the therapeutic candidates identified in this study, BRD-K57589644, Quercetin, and Catechin, were nominated through computational screening and docking simulations. No cellular or biochemical validation was performed to confirm target engagement or functional modulation. These compounds should therefore be considered computational leads for future experimental testing, not validated therapeutic agents. In future studies, rigorous experimental testing in preclinical models to confirm its efficacy, optimal dosing, and ability to penetrate the blood–brain barrier and modulate the proposed AN-associated co-expression patterns and DEK *in vivo*.

## Data Availability

All multi-omics data supporting this study are publicly accessible via three repositories: Gene Expression Omnibus (GEO, https://www.ncbi.nlm.nih.gov/geo/), IEU Open GWAS database (https://gwas.mrcieu.ac.uk/), GeneCards (https://www.genecards.org/). Analyzed datasets include GSE18838, GSE49126, GSE22491, GSE6613, GSE57475, GSE140231, and GWAS summary statistics (ieu-b-7). Analytical R scripts are available from the corresponding author upon reasonable request.
