# Supplementary material for: A neuron-distributed DEK integrates ac4C modification and neuroinflammation in pathogenesis of Parkinson disease: evidence from Mendelian randomization, multi-omics and in vitro validation
Source: Front Neurosci. 2026 Sep 1;20:1950353. doi: 10.3389/fnins.2026.1950353 (PMC13574935; doi:10.3389/fnins.2026.1950353)
Supplement: Supplementary file 1 [file Supplementary_file_1.docx]

**Figure S1: Forest plot illustration of MR inverse variance weighted results of PN-associated risk DEGs.**

**Figure S2: Pre-processing of single-cell data. (A-D)** QC metric. **(E-F)** Reduction analysis.
