# Supplementary material for: A neuron-distributed DEK integrates ac4C modification and neuroinflammation in pathogenesis of Parkinson disease: evidence from Mendelian randomization, multi-omics and in vitro validation
Source: Front Neurosci. 2026 Sep 1;20:1950353. doi: 10.3389/fnins.2026.1950353 (PMC13574935; doi:10.3389/fnins.2026.1950353)
Supplement: Supplementary file 2 [file Supplementary_file_2.docx]

**Table 1. Heterogeneity test analysis results**

| **Exposure** | **Outcome** | **gene** | **CQS** | **DCS** | **Pvalue** |
| --- | --- | --- | --- | --- | --- |
| eqtl-a-ENSG00000080824 | PD  id:ieu-b-7 | HSP90AA1 | 52.8549971138225 | 54 | ＜0.001 |
| eqtl-a-ENSG00000124795 |  | DEK | 0.419783411464665 | 8 | 0.006 |
| eqtl-a-ENSG00000138795 |  | LEF1 | 32.6708766927417 | 38 | 0.003 |
| eqtl-a-ENSG00000168310 |  | IRF2 | 11.9835170723201 | 24 | 0.048 |
| eqtl-a-ENSG00000171552 |  | BCL2L1 | 43.2695810649563 | 42 | ＜0.001 |
| eqtl-a-ENSG00000172757 |  | CFL1 | 38.8995052989616 | 50 | 0.001 |
| eqtl-a-ENSG00000186716 |  | BCR | 29.6938932976942 | 35 | ＜0.001 |

Abbreviation: CQS, Cochran’s Q statistic; DCS, Degrees of freedom of Cochran’s Q statistic; pvalue, P-value for Cochran’s Q test

**Table 2. Multilevel pleiotropy test**

| **exposure** | **outcome** | **gene** | **Pvalue** |
| --- | --- | --- | --- |
| eqtl-a-ENSG00000080824 | PD id:ieu-b-7 | HSP90AA1 | 0.87030461134286 |
| eqtl-a-ENSG00000124795 |  | DEK | 0.983944626956448 |
| eqtl-a-ENSG00000138795 |  | LEF1 | 0.624826674013202 |
| eqtl-a-ENSG00000168310 |  | IRF2 | 0.805144700046243 |
| eqtl-a-ENSG00000171552 |  | BCL2L1 | 0.384146114747399 |
| eqtl-a-ENSG00000172757 |  | CFL1 | 0.955727758128549 |
| eqtl-a-ENSG00000186716 |  | BCR | 0.57754298548484 |

**Table 3. Steiger directional test**

| **Exposure** | **outcome** | **gene** | **Pvalue** | **correct_causal_direction** |
| --- | --- | --- | --- | --- |
| eqtl-a-ENSG00000080824 | PD id:ieu-b-7 | HSP90AA1 | 0 | TRUE |
| eqtl-a-ENSG00000124795 |  | DEK | 2.88861403365072e-87 | TRUE |
| eqtl-a-ENSG00000138795 |  | LEF1 | 1.87303987280587e-240 | TRUE |
| eqtl-a-ENSG00000168310 |  | IRF2 | 0 | TRUE |
| eqtl-a-ENSG00000171552 |  | BCL2L1 | 0 | TRUE |
| eqtl-a-ENSG00000172757 |  | CFL1 | 0 | TRUE |
| eqtl-a-ENSG00000186716 |  | BCR | 0 | TRUE |
